# Evaluation of synergy between host and pathogen-directed therapies against intracellular *Leishmania donovani*

**DOI:** 10.1016/j.ijpddr.2019.08.004

**Published:** 2019-08-21

**Authors:** M. Shamim Hasan Zahid, Monica M. Johnson, Robert J. Tokarski, Abhay R. Satoskar, James R. Fuchs, Eric M. Bachelder, Kristy M. Ainslie

**Affiliations:** a Division of Pharmacoengineering and Molecular Pharmaceutics, Eshelman School of Pharmacy, University of North Carolina at Chapel Hill, Chapel Hill, NC, 27599, USA; b Department of Pathology, Medical Center, The Ohio State University, Columbus, OH, 43210, USA; c Division of Medicinal Chemistry and Pharmacognosy, College of Pharmacy, The Ohio State University, Columbus, OH, 43210, USA; d Department of Microbiology and Immunology, University of North Carolina at Chapel Hill, Chapel Hill, NC, 27599, USA

**Keywords:** Visceral leishmaniasis, Resazurin assay, Host-directed therapy, Combinatorial therapy, AR-12, DNER-4

## Abstract

Visceral leishmaniasis (VL) is associated with treatment complications due to the continued growth of resistant parasites toward currently available pathogen-directed therapeutics. To limit the emergence and combat resistant parasites there is a need to develop new anti-leishmanial drugs and alternative treatment approaches, such as host-directed therapeutics (HDTs). Discovery of new anti-leishmanial drugs including HDTs requires suitable *in vitro* assay systems. Herein, we modified and evaluated a series of resazurin assays against different life-stages of the VL causing parasite, *Leishmania donovani* to identify novel HDTs. We further analyzed the synergy of combinatorial interactions between traditionally used pathogen-directed drugs and HDTs for clearance of intracellular *L. donovani*. The inhibitory concentration at 50% (IC_50_) of the five evaluated therapies [amphotericin B (AMB), miltefosine, paromomycin, DNER-4, and AR-12 (OSU-03012)] was determined against promastigotes, extracellular amastigotes, and intracellular amastigotes of *L. donovani* via a resazurin-based assay and compared to image-based microscopy. Using the resazurin-based assay, all evaluated therapies showed reproducible anti-leishmanial activity against the parasite's different life-stages. These results were consistent to the traditional image-based technique. The gold standard of therapy, AMB, showed the highest potency against intracellular *L. donovani*, and was further evaluated for combinatorial effects with the HDTs. Among the combinations analyzed, pathogen-directed AMB and host-directed AR-12 showed a synergistic reduction of intracellular *L. donovani* compared to individual treatments. The modified resazurin assay used in this study demonstrated a useful technique to measure new anti-leishmanial drugs against both intracellular and extracellular parasites. The synergistic interactions between pathogen-directed AMB and host-directed AR-12 showed a great promise to combat VL, with the potential to reduce the emergence of drug-resistant strains.

## Highlights

• An inexpensive and simple modified resazurin drug screening assay for *Leishmania donovani.*
• Anti-leishmanial therapies are evaluated in axenic amastigotes, promastigotes, and intracellular *Leishmania donovani.*
• Identification of synergistic, additive, and antagonistic activity between therapies.
• Amphotericin B and host-directed AR-12 had significant synergistic anti-leishmanial activity.

## Graphical abstract

**Figure:**
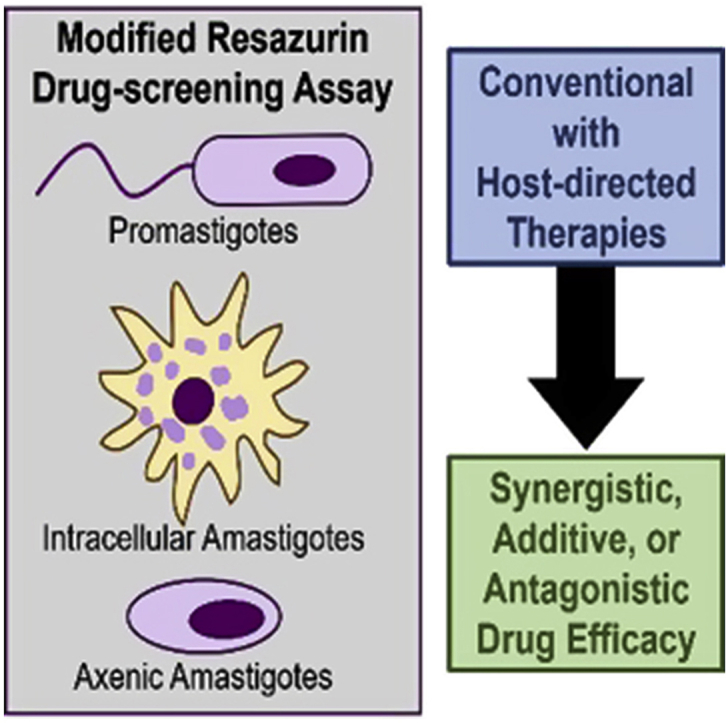

## 1 Introduction

Leishmaniasis is a life-threatening neglected tropical disease caused by obligate intracellular parasites of the genus *Leishmania* and approximately a tenth of the world is at risk of infection (World Health Organization, 2018; Zulfiqar et al., 2017). The disease is broadly classified as either a cutaneous (CL) or visceral leishmaniasis (VL), the latter being the disease's most fatal form and is mainly caused by two species (of 29 species): *Leishmania donovani* (*L. donovani*) and *L. infantum*. Globally, ∼90,000 new cases of VL are reported each year, with an approximately 95% fatality rate if left untreated (World Health Organization, 2018; Zulfiqar et al., 2017).

A vaccine against *Leishmania* has not yet been developed and current first-line therapies include amphotericin B (AMB), miltefosine, paromomycin, and antimonials [e.g., sodium stibogluconate (SSG)] (Palatnik-de-Sousa, 2008). These therapies have adverse side effects, often require long treatment regimens, and act directly on the pathogen, imposing an increased risk of developing drug resistance (Croft et al., 2006; Freitas-Junior et al., 2012; Sundar and Chakravarty, 2010). Drug resistance was presented in more than 60% of clinical isolates in the Bihar region of India, with reported resistance to SSG (Rijal et al., 2003), and AMB (Purkait et al., 2012). For example, the parasite *Leishmania* are prone to acquiring resistance to miltefosine due to its elongated drug half-life (∼150 h), long treatment course (∼28 days), and parasite susceptibility to develop a single point mutation (Mishra and Singh, 2013; Perez-Victoria et al., 2006; Seifert et al., 2007).

New approaches to traditional monotherapies are needed to combat drug resistance. Combinatorial therapies could decrease monotherapy duration and dosage, leading to reduced resistance (Sundar and Chakravarty, 2013). A reduced treatment duration was observed in a clinical trial using paromomycin and SSG to cure VL. The treatment duration went from 30 days with monotherapy to 17 days with combination (Musa et al., 2012). In another clinical study, a single dose of liposomal AMB (AmBisome) with 7-days of miltefosine cured 98% of the VL patients, compared to 91% with AMB alone (Sundar et al., 2008). However, there is no evidence suggesting that combinatorial effects of two primarily pathogen-mediated drugs mitigates drug-resistance.

In addition to combined therapies, host-directed therapies (HDTs) may better target the host's response to the pathogen as opposed to just the pathogen directly, which could lead to reduced emergence of resistance (Collier et al., 2013). Often HDTs' mechanism is to modulate the host's immune response through induction of pro-inflammatory cytokines (e.g., IFN-γ, IL-12). In particular, IFN-γ has been shown to be essential in treating leishmaniasis (Wang et al., 1994). Combination treatment with pentavalent antimonials and recombinant IFN-γ increased the VL cure rate compared to individual treatment alone (Squires et al., 1993; Sundar et al., 1997). Similarly, sub-optimal doses of AMB with IL-12 cleared VL compared to higher dosing of AMB alone (Murray et al., 2003). Although promising, the high cost of recombinant protein and required cold-chain storage reduces the feasibility of cytokine therapies in developing nations.

There are a few small molecule HDTs to treat *Leishmania* currently in the development pipeline, and they may work synergistically in combination with pathogen-directed therapies (i.e., AMB, miltefosine). Celecoxib, a Cyclooxygenase-2 (COX-2) inhibitor, has been shown to suppress tumor cell viability through disruption of PDK-1/Akt signaling and induction of apoptosis (Kucab et al., 2005; Zhou et al., 2018). Our laboratory has evaluated AR-12 (OSU-03012), an IND-approved derivative of celecoxib that lacks COX-2 inhibitor activity, as a novel HDT against intracellular *L. donovani* (Collier et al., 2016). In addition, AR-12 has been reported to induce host-mediated reduction of *Salmonella enterica* (Chiu et al., 2009a; Hoang et al., 2014), *Francisella tularensis* (Hoang et al., 2016), *F. novicida* (Chiu et al., 2009b), and *Cryptococcas neoformans* (Baxter et al., 2011). Although promising, AR-12's hydrophobicity makes it difficult to deliver at therapeutic levels. To deliver it better, we have encapsulated AR-12 within biodegradable acetalated dextran (Ace-DEX) microparticles (AR-12/MPs), which can passively target phagocytic host cells for site-specific drug delivery (Collier et al., 2016; Hoang et al., 2014, 2016). We have previously shown that treatment with AR-12/MPs significantly reduced hepatic, splenic, and bone marrow *L. donovani* loads in infected mice compared to free AR-12 (Collier et al., 2016).

In addition to AR-12, our group has aided in discovery of anti-leishmanial compounds isolated from the roots of *Pentalinon andrieuxii*, including pentalinonsterol (PEN) and 6,7-dihydroneridienone (DNER), both of which have host-mediated as well as pathogen-directed activity. Although not fully elucidated, as derivatives of plant sterols, both PEN and DNER could interfere with the sterol biosynthesis pathway (Pan et al., 2012). Suboptimal doses of DNER has anti-leishmanial activity (IC_50_ = 1.4 μM) against intracellular *L. mexicana*, in addition to direct activity against promastigotes at higher concentrations (IC_50_ = 9.2 μM) (Pan et al., 2012). Herein, we are evaluating the effects of a DNER analog, DNER-4, against *L. donovani*.

The discovery of new anti-leishmanial compounds largely depends on a simple, cheap, and reproducible assay system; however, they are often difficult to develop because the parasite has a complex life-cycle, requiring drug screening against promastigotes, and extracellular and intracellular amastigotes (Callahan et al., 1997; Kamhawi, 2006; Kiderlen and Kaye, 1990; Mikus and Steverding, 2000). For evaluation of intracellular amastigotes, labor-intensive microscopy-based direct counting of cells and parasites is considered the gold-standard (Neal and Croft, 1984). For both life-stages, reporter gene based automated screening is also not ideal as most assays require drug selection for maintaining the optimal reporter gene expression, which could directly interfere with the anti-infective efficacy, increasing false positives (Mandal et al., 2009). Moreover, there are issues of sensitivity and background in some reporter gene-based assays as they often cannot differentiate live and dead intracellular amastigotes (Gupta, 2011; Sereno et al., 2007). A resazurin based assay could be better because a metabolically active parasite is required to form fluorescent resorufin, and it can be analyzed in a throughput manner irrespective of a genetically modified parasites, which can facilitate use of clinical isolates (Paape et al., 2014). However, due to potential host cell interference, resazurin-based anti-leishmanial drug screening in an intracellular system requires some modifications. This article modifies and evaluates a resazurin based drug screening assay using different *L. donovani* life-stages to identify new anti-leishmanial combination therapies of conventional pathogen-directed drugs and host-directed compounds (AR-12 and DNER-4).

## 2 Materials and methods

### 2.1 Chemicals and reagents

All reagents were purchased from Sigma-Aldrich (St. Louis, MO) unless indicated otherwise. AR-12 was purchased from GenDEPOT (Katy, TX). DNER-4 is an analog of plant sterol DNER derived from cholic acid. The synthetic procedures for the preparation of DNER-4 and associated characterization data are included in the Supplementary Information (SI) file.

### 2.2 Compound mammalian cytotoxicity determination

Mouse bone marrow-derived macrophages (BMDMs) isolation from BALB/c mice and culture was done as described previously (Weischenfeldt and Porse, 2008), and approved by the Animal Care and Use Committees at UNC. A lactate dehydrogenase (LDH) assay (Thermo Fisher Scientific, Grand Island, NY) was used to determine cytotoxicity per manufacturer's directions. Control wells had DMEM or DMEM containing 0.1% dimethyl sulfoxide (DMSO).

### 2.3 Resazurin-based drug susceptibility assay on promastigotes and axenic amastigotes

*Leishmania donovani* strain (ATCC 30030; ATCC; Manassas VA) was used in all studies and grown via manufacture's specification. The promastigotes screening were performed as described previously, with few modifications (Kulshrestha et al., 2013). Late log-phase promastigotes (1 × 10^5^ per one 96-well) were incubated with drug for 72 h at 25 °C. Resazurin was added [0.002% (w/v)], incubated for 24 h and assessed fluorometrically (ex: 544 nm/em: 590 nm; SpectraMax M2, Molecular Devices, Sunnyvale, CA). *L. donovani* axenic amastigotes were differentiated as described previously, with little modification (Ephros et al., 1997). Late log-phase promastigotes were differentiated into axenic amastigotes in a low pH (6.3) medium for 96 h at 37 °C. Fully differentiated axenic amastigotes (2 × 10^5^ per one 96-well) were incubated 72 h with drug at 37 °C, and resazurin assay was then performed as indicated above. The parasite's Inhibitory Concentration 50% (IC_50_) was determined from a best fit trend-line of two experimental repeats.

### 2.4 Image-based evaluation of intracellular anti-leishmanial activity

An image-based anti-leishmanial assay was used as described previously with slight modification (Collier et al., 2016). BMDMs seeded on round glass coverslips (5 × 10^5^ cells per one 24-well) were allowed to adhere overnight, infected with stationary phase *L. donovani* promastigotes (MOI 1:10), incubated overnight, and washed (3×) with warmed serum-free media. Compounds were added, incubated for 72 h, washed (3×) with PBS, fixed with methanol and stained with Giemsa stain (1:20 in H_2_O). An EVOS XL (100×, Thermo Fisher Scientific) was used for imaging. *L. donovani* amastigotes per 100 macrophages was determined, in duplicate, and in a blinded fashion.

### 2.5 Resazurin-based viability analysis of L. donovani in infected macrophages

BMDMs (1 × 10^5^ per one 96-well) infected overnight with stationary phase *L. donovani* promastigotes (MOI 1:10), were washed (3×) with serum free DMEM, and incubated with compounds for 72 h. Then, parasites were rescued with BMDM controlled lysis, modified from a previous report (Kiderlen and Kaye, 1990). In brief, cells were washed with serum-free HEPES-buffered RPMI (HEPES-RPMI; 1×), lysed (20 min) with HEPES-RPMI (100 μL/well) with 0.008% SDS (w/v), SDS neutralized with HEPES-RPMI with 17% FBS (v/v; 150 μL/well) and placed in an air-tight container (25 °C for 96 h). Resazurin [0.02% (w/v); 25 μL] was added, incubated (24 h) and was assessed fluorometrically.

### 2.6 Determination of intracellular anti-leishmanial synergy

The combination index (CI) for each of the combinations of the drugs was determined by using the Chou et al. method using Eqn (1):

(1) $$\text{CI}=\frac{C_{A}}{{\text{IC}}_{50A}}+\frac{C_{B}}{{\text{IC}}_{50B}}$$

where *C*_*A*_ and *C*_*B*_ are the concentrations of the corresponding drugs, and IC_50*A*_ and IC_50*B*_ are the IC_50_ values of the drugs when administrated alone (Chou and Talalay, 1984).

## 3 Results and discussion

### 3.1 Validation of resazurin assay for anti-leishmanial drug screening

**Fig. 1:**
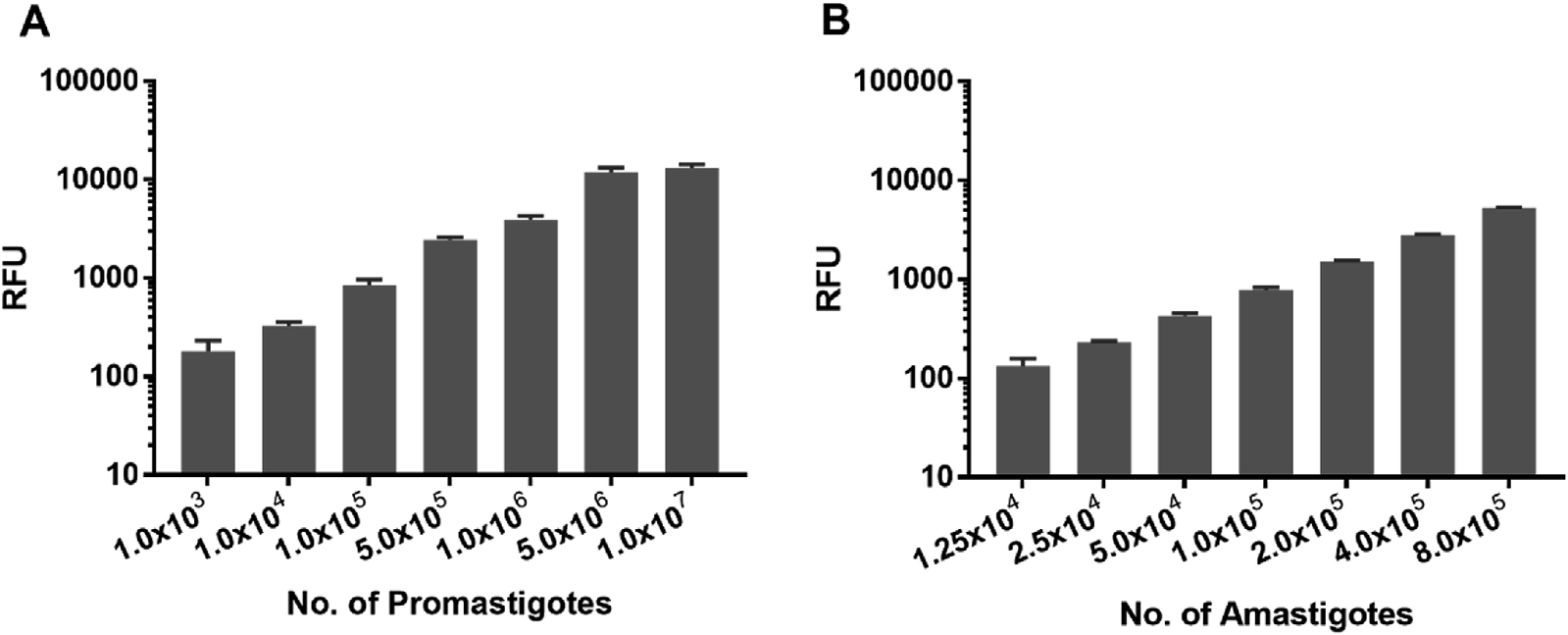
Resazurin assay to measure *L. donvovani* (A) promastigote and (B) amastigote viability. Correlation between Relative Fluorescence Units (RFUs) production with different parasite numbers were highly significant (*p* < 0.005) as determined by Pearson's r-analysis (*r* > 0.94 for promastigotes, and *r* > 0.99 for amastigotes). This is a representative data of two biological repeats, which is reported as mean ± standard deviation (SD) of triplicate samples and with background subtracted.

Resazurin, a blue non-fluorescent and non-toxic dye is irreversibly reduced to a pink, highly fluorescent resorufin by mitochondrial dehydrogenase enzymes within metabolically active cells (Shahangian et al., 1984). Although resorufin can be further reduced to nonfluorescent dihydroresorufin, a linear increase of fluorescent signal was observed with viable *Leishmania* parasites over a period of 72 h, demonstrating the stability of resorufin–mediated signal (Paape et al., 2014). In addition to its utility in confirming microbiological contamination in milk, resazurin-based reduction assay is widely used to analyze chemical cytotoxicity and minimum inhibitory concentration values for antimicrobial agents (Hudman and Sargentini, 2013; McNicholl et al., 2007; O'Brien et al., 2000; Sarker et al., 2007). We have evaluated resazurin-based anti-leishmanial drug screening assay on different life-stages of parasite because it is simple, cost-effective, reproducible and requires a viable parasite. This assay is relatively inexpensive (∼$13/gram of resazurin) and can be used in a throughput screening manner with wild-type strains. Other screening assay, such as fluorescent-based reporter genes and monoclonal antibodies for flow cytometry are less sensitive and have limitations for longer drug treatment duration (Abdullah et al., 1999). Additionally, reporter gene-based screening requires genetically modified parasites, limiting evaluation against clinical strains (Mandal et al., 2009). Moreover, fluorescent markers can have a high background due to auto-fluorescence of host cells and cannot distinguish between live and dead intracellular amastigotes, which affects both the reproducibility and sensitivity of these assays (Gupta, 2011; Sereno et al., 2007). Fig. 1 reports the validation of resazurin assay with extracellular promastigotes and amastigotes (Kulshrestha et al., 2013; Paape et al., 2014) by confirming an increase in parasite number with Relative Fluorescence Units (RFUs). A direct significant linear correlation (*p* < 0.005) between conversion of resazurin to RFUs with increasing parasite numbers were observed in Pearson's r-analysis (*r* > 0.94). Moreover, promastigote and amastigote viability testing with resazurin is highly reproducible, and required 10^3^ viable *Leishmania* parasites to generate a detectable fluorescent signal in our experimental set up (Fig. S1). On the contrary, dead *Leishmania* parasites (treated with 10% SDS for 20 min to ensure complete death) did not produce greater fluorescent signal compared to media background (data not shown) demonstrated resazurin assay is highly sensitive to detect viable parasites.

**Fig. 2:**
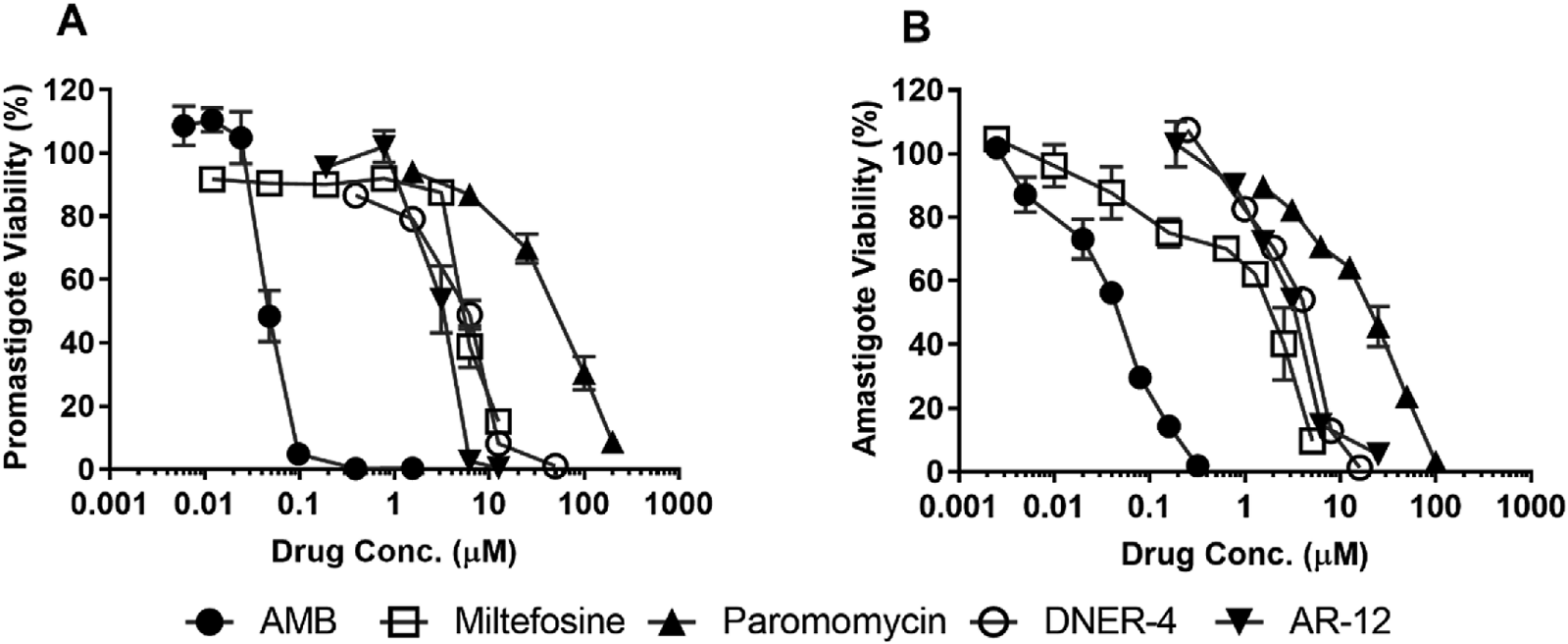
Direct effects of drugs on (A) extracellular promastigotes and (B) axenic amastigotes, via resazurin assay. Data (mean ± SD) are normalized to non-treated control groups. Assays ran in triplicate with two biological repeats.

For anti-leishmanial drug screening, promastigote viability is often used, but it is not always reproducible in an intracellular infection model (De Muylder et al., 2011). Axenic amastigotes may better replicate intracellular models (Sereno and Lemesre, 1997). Herein, known and potential anti-leishmanial compounds were evaluated against promastigotes and axenic amastigotes using a resazurin assay (Fig. 2, Table 1). Among the current therapies, AMB was the most potent against both promastigotes (IC_50_ 0.047 μM) and axenic amastigotes (IC_50_ 0.049 μM) with miltefosine and paromomycin as the most effective against agent axenic amastigotes. The IC_50_s of AR-12 and DNER-4 did not vary much across promastigote (3.3 and 5.82 μM, respectively) and axenic amastigote (3.45 and 4.45 μM, respectively) stages.

**Table 1:** Anti-leishmanial drugs against extracellular promastigotes, amastigotes, and intracellular amastigotes. All assays were run in triplicate and data (means ± SD) from two biological repeats. IC_50_: Inhibitory Concentration at 50%; LD_50_: 50% Lethal Dose for BMDMs. *, significant lower IC_50_ of drugs against intracellular amastigotes compared to extracellular promastigotes of the respective group (**p < 0.01, ***p < 0.001, ****p < 0.0001).^#^, significant lower IC_50_ of drugs against intracellular amastigotes compared to axenic amastigotes of the respective group (^###^*p* < 0.001).^‡^, significant lower IC_50_ of drugs against axenic amastigotes compared to extracellular promastigotes of the respective group (^‡^*p* < 0.01,^‡‡^*p* < 0.001). A one-way ANOVA (Tukey's multiple comparisons test) was conducted comparing effects of the drugs on different life-stages of the parasites.

Compounds	IC_50_ (μM) for *L. donovani*				Cytotoxicity LD_50_ (μM)
	Extracellular promastigotes	Axenic amastigotes	Intracellular amastigotes		
			Image based assay	Resazurin assay	
Amphotericin B	0.047 ± 0.004	0.049 ± 0.005	0.024 ± 0.001	0.033 ± 0.011	38.5 ± 4.95
Miltefosine	5.4 ± 0.42	2.13 ± 0.53^‡^	1.1 ± 0.37	0.55 ± 0.15****^/###^	62.5 ± 4.95
Paromomycin	59 ± 5.66	22 ± 4.24^‡‡^	15.2 ± 4.67	11.6 ± 0.42****	>800
DNER-4	5.82 ± 1.10	4.45 ± 0.21	5.2 ± 2.55	3.2 ± 0.28**	78.5 ± 9.2
AR-12	3.3 ± 0.42	3.45 ± 0.07	1.18 ± 0.18	1.44 ± 0.08***^/###^	11.1 ± 2.5

There are significant differences that exist between intracellular amastigotes and axenic amastigotes in protein expression and drug susceptibility (Holzer et al., 2006; Pescher et al., 2011). Spleen-derived hamster *L. donovani* amastigotes were more infective and pathogenic compared to axenically grown amastigotes due to overexpression of virulence associated proteins (Iyer et al., 2008; Pescher et al., 2011). In another study, anti-leishmanial activity of naloxonazine was found only against intracellular *L. donovani* amastigotes but not on axenically grown amastigotes (De Muylder et al., 2011). These studies highlight the importance of evaluating anti-leishmanial drugs against promastigotes, axenic amastigotes and intracellular amastigotes and therefore drugs were screened against intracellular amastigotes using the modified resazurin assay.

**Fig. 3:**
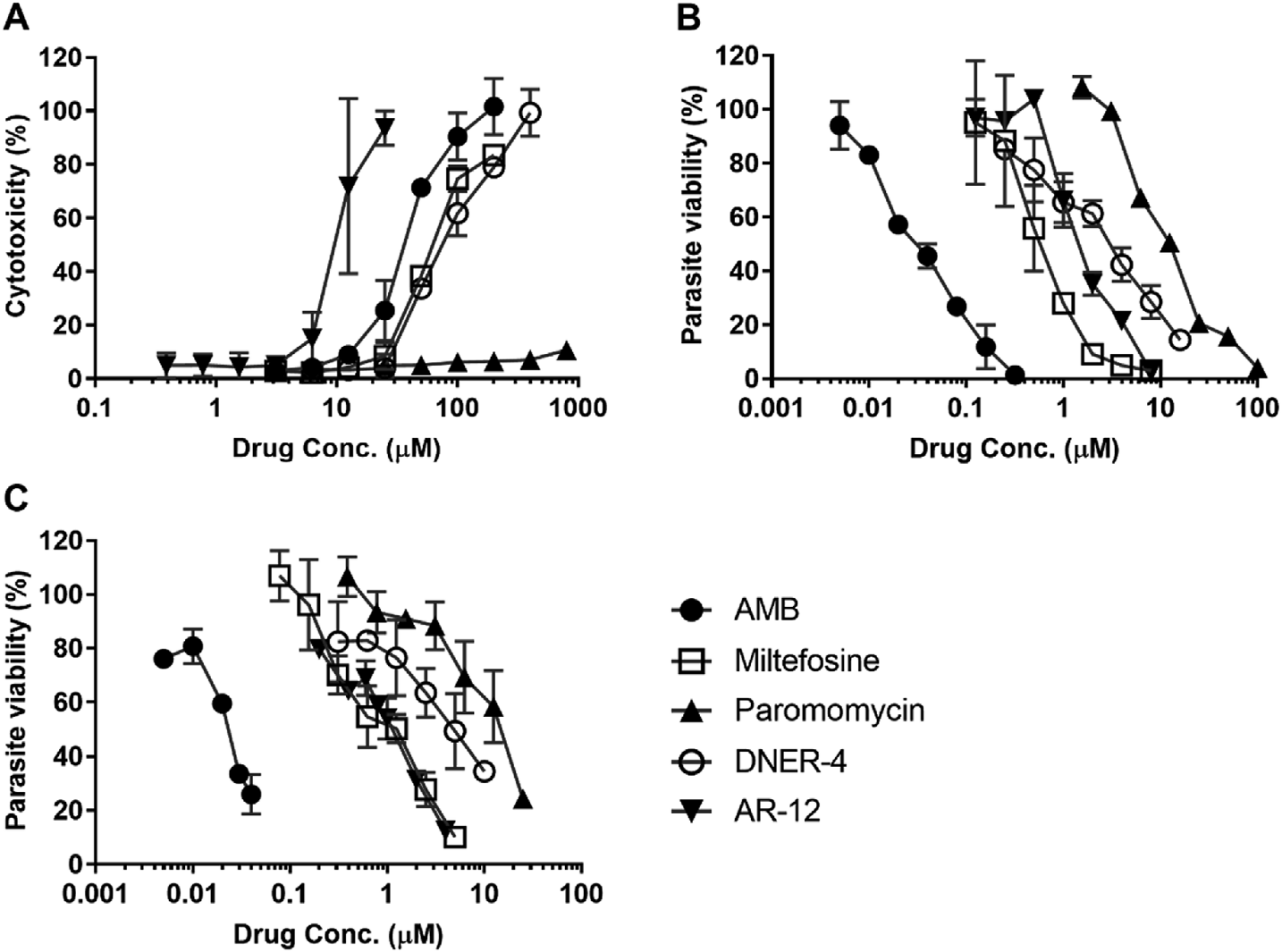
(A) Macrophage cytotoxicity, and intracellular parasite burden in presence of drugs (normalized to untreated), determined by (B) the modified resazurin assay and (C) the traditional microscopic image-based assay. Data is presented as means ± SD, ran in triplicate with two biological replicates.

The relative cytotoxicity of the evaluated compounds was analyzed on mouse BMDMs (Fig. 3A, Tables 1 and S1). Using a non-toxic dosing range, the modified resazurin-based method was used for determination of intracellular anti-leishmanial activity (Table 1, Fig. 3B). These results were comparable to the traditionally used microscopic technique (Table 1, Figs. 3C and S2), suggesting that the modified resazurin assay can be used in place of labor-intensive, microscopy-based direct counting method.

The IC_50_s of AMB did not differ much across extracellular promastigote (0.047 μM), axenic amastigote (0.049 μM) and intracellular amastigotes (0.033 μM), as previously reported (Paape et al., 2014). However, miltefosine and paromomycin showed greater intracellular anti-leishmanial activity compared to extracellular anti-infectivity, indicating potential host-directed activity (Table 1). Miltefosine not only acts directly on the cell membrane of *Leishmania*, but it can also induce IFN-γ receptor activity in *L. donovani*-infected macrophages, which might contribute to the host-directed effects (Wadhone et al., 2009). The intracellular anti-leishmanial activity of HDT AR-12 presented here (Fig. 3, Table 1) corresponds with our published work where the IC_50_ of extracellular promastigotes and amastigotes (3.3 and 3.45 μM, respectively) are significantly higher than intracellular concentrations (1.44 μM), supporting a host-mediated mechanism (Collier et al., 2016). Although not fully elucidated, AR-12 mediated inhibition of Akt kinase signaling could modulate *Leishmania* inducing Akt signaling to confer host cell resistance to apoptosis (Kucab et al., 2005; Neves et al., 2010; Ruhland et al., 2007). Hence, it is possible that AR-12 may interfere with the parasite-mediated induction of Akt signaling. Future work will aim to identify the mechanism of AR-12 mediated clearance of intracellular *L. donovani*.

DNER-4 also showed potential direct activity on extracellular promastigotes and amastigotes (5.82 and 4.45 μM, respectively) (Table 1); however, it was more potent against the intracellular parasite (3.2 μM), indicating a preference in host-directed activity. Although not fully elucidated, DNER-4 could interfere with the sterol biosynthesis pathway of *Leishmania*, which would disrupt the normal structure and function of parasites (de Souza and Rodrigues, 2009; Pan et al., 2012). On the other hand, several phytosterols, such as sitosterol can induce IFN-γ production in human peripheral blood mononuclear cells (Brull et al., 2010). Thus, as a derivative of a plant sterol, DNER-4 could induce IFN-γ production in mouse BMDMs to attribute to its host-directed leishmanicidal activity. However, further studies are warranted to confirm these hypotheses.

### 3.2 Combinatorial host-directed leishmanicidal activities of anti-leishmanial drugs

**Fig. 4:**
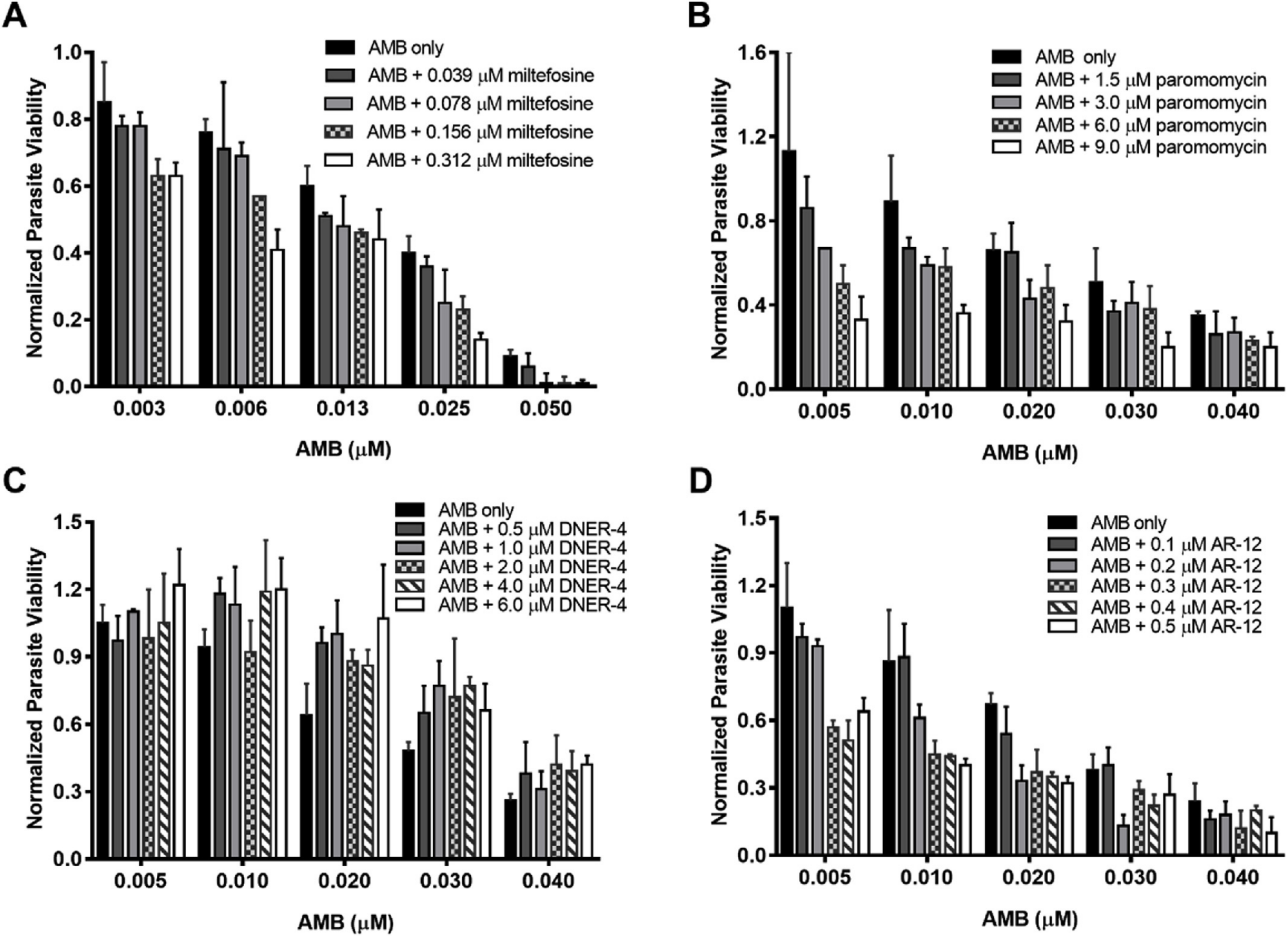
Intracellular anti-leishmanial efficacy (normalized to untreated infected control) of amphotericin B (AMB) in combination with other drugs: (A) miltefosine, (B) paromomycin, (C) DNER-4, and (D) AR-12 via resazurin assay. Data is presented as means ± SD, ran in triplicate with two biological replicates.

**Fig. 5:**
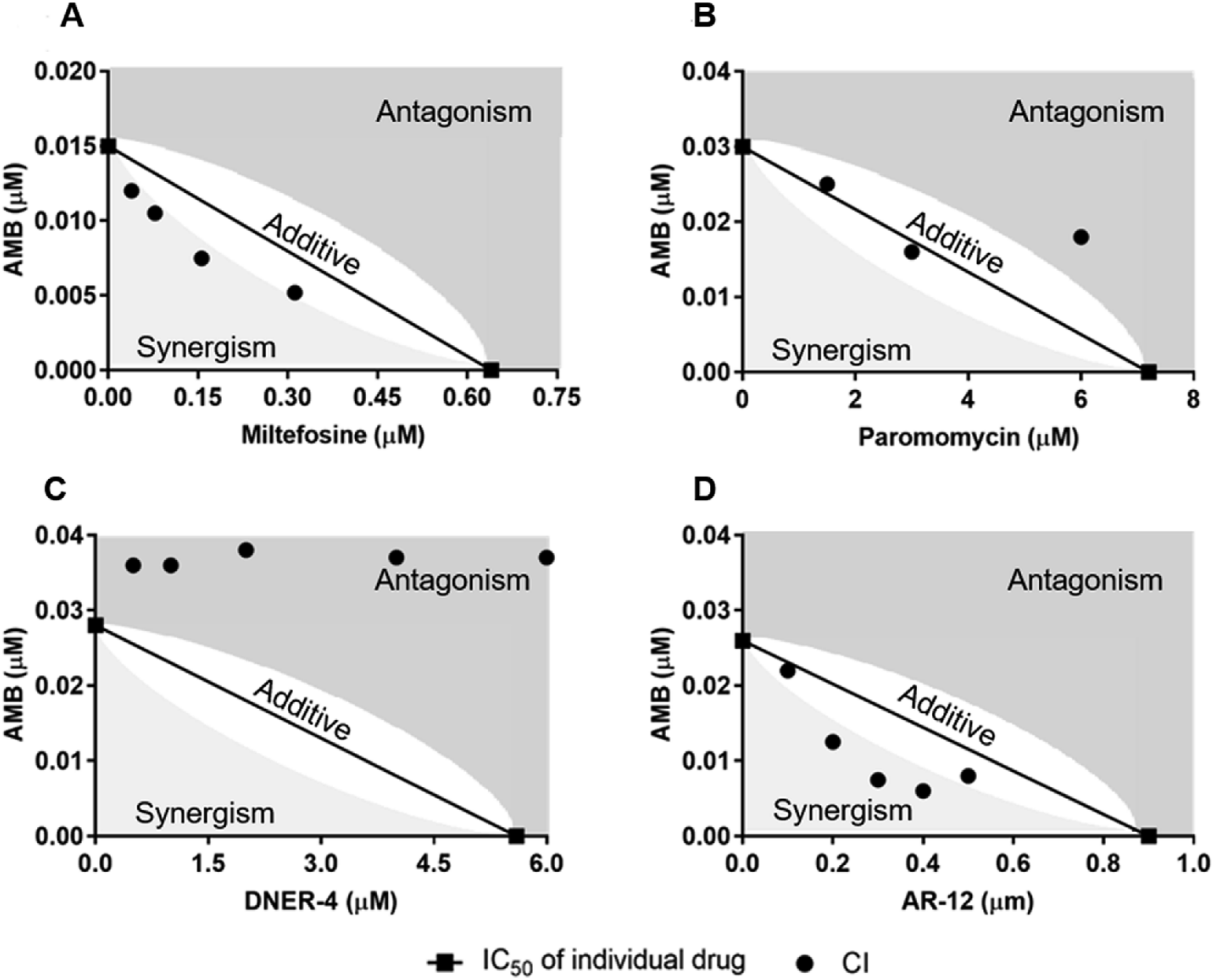
Isobolograms demonstrating combinatorial effects of amphotericin B (AMB) with other anti-leishmanial drugs (normalized to untreated controls): (A) miltefosine, (B) paromomycin, (C) DNER-4, and (D) AR-12 via resazurin assay. A combination index (CI) = 1 of drugs represented additivity line, and the error range of additivity is accompanied with a clear window. CI values above the error range of additivity line (in the dark grey shaded region) represented antagonistic effects (CI > 1) between drugs, and points below the error range of the additivity line reflected synergistic effects (CI < 1, light grey).

We aimed to evaluate the cominbation therapy of two anti-leishmanial drugs utilzing the modified resazurin assays. Irrespective of the parasitic life-stages, AMB was the most potent (i.e., lowest IC_50_) among the tested compounds (Table 1). A combinatorial assay was performed varying AMB concentration at fixed and sub-optimal concentrations of other compounds (Fig. 4). Normalized parasite viability with comparison to untreated controls was calculated for individual drugs as well as for their combinations. However, to better highlight the synergistic effects of the combination therapies depicted in Fig. 4, isobolograms were generated for each of the combinations (Fig. 5). The combination index (CI) was determined for each of the combinations of the drugs by using Eqn (1). As shown in Fig. 5, the additivity line (CI = 1) is accompanied with a clear window represents the error range of additivity. Thus, points above the additivity line (CI > 1, dark grey) are antagonistic, and those below the line (CI < 1, light grey) are synergistic.

AMB and miltefosine demonstrated mild synergistic effects (Fig. 5A), although it has been previously reported that the anti-leishmanial activity of miltefosine was greatly enhanced in combination with AMB *in vivo* (Seifert and Croft, 2006). As shown in Fig. 5B, paromomycin in combination with AMB showed additive anti-leishmanial interactions at relatively lower doses, however, at higher concentrations, antagonism was observed. Although primarily pathogen-directed therapies like AMB, miltefosine, and paromomycin can effectively cure VL, it is unlikely that these drugs (either individual or in combination) could mitigate the emergence of drug-resistant strains.

To further improve the current combinatorial approach to treat VL, we analyzed the combinatorial effects between a pathogen-directed and a HDT. Therefore, we tested the effects of DNER-4, a potential HDT, in combination with AMB against intracellular *L. donovani.* As outlined in Fig. 5C, a strong antagonistic effect was observed when combined with AMB. This antagonistic response could be due to the affinity of AMB for sterols or sterol derivatives like DNER-4 (Pan et al., 2012; Urbina et al., 1987). It can be hypothesized that AMB interacts with DNER-4, diminishing the leishmanicidal properties of both drugs in a combined therapy. These results highlight the importance of evaluating the chemical properties and mechanisms of action of individual drugs when selecting for a combination therapy.

**Fig. 6:**
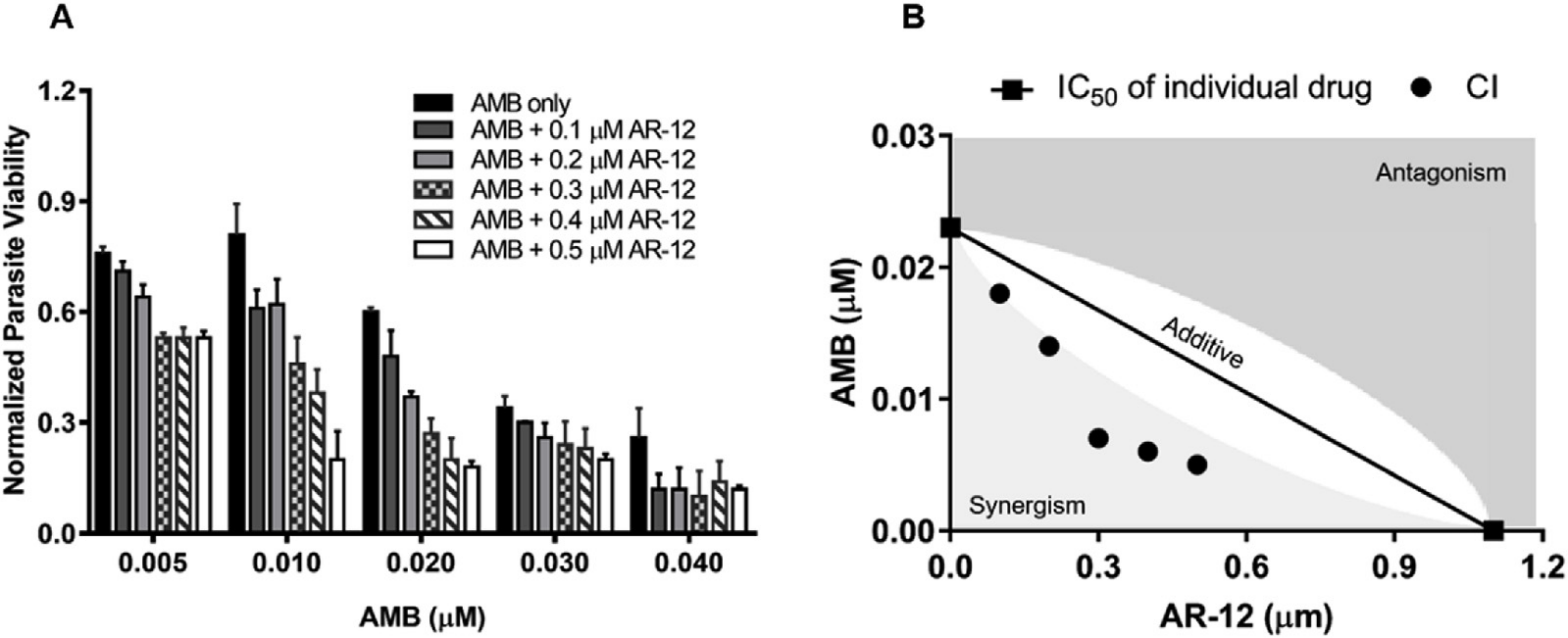
Combinatorial effects were determined by traditional Giemsa stain-based image assay. (A) Anti-leishmanial effects of AMB and AR-12. (B) Isobologram showing combinatorial interactions between AMB and AR-12.

Next, we evaluated the combinatorial effects of the host-directed drug AR-12 with AMB to eradicate intracellular *Leishmania*. Herein, we demonstrate that a synergistic effect, indicative of CI < 1, was observed when variable suboptimal concentrations of AMB in combination with suboptimal doses of AR-12, compared to AMB treatment alone (Fig. 5D and Table 2). These results were confirmed using the traditional image-based counting of amastigotes around BMDM nuclei (Fig. S3). Image-based evaluation of reduced parasite viability (Fig. 6A) and synergistic CI values (Fig. 6B) with combinatorial treatment of suboptimal doses of AMB and AR-12 compared to either treatment alone further demonstrated the anti-leishmanial efficacy of this combination (Table 2).

**Table 2:** Combination index (CI) for the synergistic effects of AMB and AR-12. Data were performed in triplicate and reported as mean.

AR-12 (μM)	Resazurin assay		Image-based assay	
	IC_50_ of AMB (μM)	CI	IC_50_ of AMB (μM)	CI
0	0.033	–	0.024	–
0.1	0.022	0.96	0.018	0.85
0.2	0.013	0.70	0.014	0.79
0.3	0.008	0.62	0.007	0.56
0.4	0.006	0.66	0.006	0.62
0.5	0.008	0.84	0.005	0.68

Synergistic intracellular leishmanicidal interaction between pathogen-directed AMB and host-directed AR-12 showed great promise to treat VL. To truly evaluate the effects of synergistic pathogen and host-directed therapies, additional studies will need to be performed to evaluate both *in vitro* and *in vivo* synergistic anti-leishmanial interactions of AMB and AR-12 on a drug resistant *L. donovani.* However, prior to evaluating this therapy *in vivo,* limitations of toxicity and hydrophobicity of the two drugs needs to be addressed. For AR-12, these concerns can be mitigated by formulating AR-12 into biodegradable Ace-DEX microparticles (Ace-DEX MPs), as we showed previously (Collier et al., 2016). Additionally, encapsulation of AMB in a liposome (Ambisome) has been shown to mitigate toxicity (Adler-Moore and Proffitt, 2002). It would be pertinent to investigate such formulations to further develop these therapies to treat *L. donovani* infections *in vivo*.

## 4 Conclusions

We have modified and evaluated resazurin-based assays on different life-stages of *Leishmania*, which will facilitate *in vitro* screening of new anti-leishmanial therapies, particularly HDTs. Modified resazurin assay for anti-leishmanial drug screening on clinically relevant intracellular amastigotes is simple, inexpensive, reproducible, and comparable to the traditional microscopy-based image assay. Also, the synergistic interactions between pathogen-directed AMB and host-directed AR-12 to clear intracellular *L. donovani* shows great promise to combat VL, particularly in view of growing resistance to available therapies. Overall, this study provides sustained efforts to enrich current pipeline of anti-leishmanial drugs and contributes to improve or modify the current therapeutic approaches to combat VL.

## Funding

This study was supported by the National Institutes of Health (NIH), United States (5R21AI123692).

## Conflicts of interest

None to declare.
