# Supplementary material for: Evaluation of synergy between host and pathogen-directed therapies against intracellular Leishmania donovani
Source: Int J Parasitol Drugs Drug Resist. 2019 Aug 21;10:125–32. doi: 10.1016/j.ijpddr.2019.08.004 (PMC6731340; doi:10.1016/j.ijpddr.2019.08.004)
Supplement: Multimedia component 1 [file mmc1.docx]

**Supplementary Information (SI)**

**Evaluation of synergy between host and pathogen-directed therapies against intracellular *Leishmania donovani***

M. Shamim Hasan Zahid, Monica M. Johnson, Robert J. Tokarski II, Abhay R. Satoskar, James R. Fuchs, Eric M. Bachelder, Kristy M. Ainslie^*^

***DNER-4 Experimental Data***

**3α,12α-diacetoxy-5β-cholan-24-oic acid methyl ester (DNER-4)**(do Nascimento et al., 2015)**:** Deoxycholic acid (1.0 g, 2.55 mmol) was dissolved in methanol (140 mL, 0.02 M). Sulfuric acid (1.0 mL) was then added slowly. The resulting reaction mixture was allowed to stir at 65° C for 24 h. After completion of the reaction, the solvent was evaporated under reduced pressure. Water was added and the mixture was extracted with DCM (3 x 50 mL). The organic extracts were combined, washed with water, dried over anhydrous Na_2_SO_4_, and evaporated under reduced pressure. The crude methyl deoxycholate was carried on to the next step without further purification. The crude compound was isolated as a white foam (0.82 g, 79% yield. ^1^H NMR (300 MHz, CDCl_3_) δ 3.98 (s, 1H), 3.66 (s, 3H), 3.64 – 3.53 (m, 1H), 0.97 (d, J = 6.3 Hz, 3H), 0.91 (s, 3H), 0.68 (s, 3H). ^13^C NMR (75 MHz, CDCl_3_) δ 174.78, 73.10, 71.66, 51.52, 48.23, 47.26, 46.54, 42.17, 36.44, 36.09, 35.38, 35.30, 34.18, 33.63, 31.20, 30.99, 30.44, 28.71, 27.57, 27.24, 26.22, 23.76, 23.19, 17.30, 12.76. NMR data obtained for this compound was in agreement with previously reported literature (He et al., 2017).

The crude methyl deoxycholate (500 mg, 1.23 mmol, 1.0 equiv.) was dissolved in pyridine (2.46 mL, 0.5 M). Acetic anhydride (4.90 mL, 0.25 M) and DMAP (75.13 mg, 0.615 mmol) were then added sequentially and the reaction mixture was allowed to stir at 25° C for 24 h. The reaction was then quenched with saturated CuSO_4_ solution and extracted with ethyl acetate (3 x 50 mL). The combined organic extracts were then washed with water (3 x 100 mL) and brine (1 x 100 mL), dried over anhydrous Na_2_SO_4,_ and concentrated under reduced pressure to give the crude product as a colorless oil. Flash column chromatography (20:80 ethyl acetate:hexanes) provided DNER-4 as a white foam (272.7 mg, 45% yield). IR (neat) 2498, 2869, 1737, 1449, 1378, 1364, 1245, 1028 cm^-1^; ^1^H NMR (300 MHz, CDCl_3_) δ 5.03 – 4.96 (m, 1H), 4.68 – 4.54 (m, 1H), 3.57 (s, 3H), 2.01 (s, 3H), 1.94 (s, 3H), 0.82 (s, 3H), 0.72 (d, *J* = 6.2 Hz, 3H), 0.64 (s, 3H). ^13^C NMR (75 MHz, CDCl_3_) δ 174.41, 170.36, 170.28, 75.79, 74.08, 51.38, 49.40, 47.51, 44.97, 41.79, 35.63, 34.69, 34.64, 34.35, 33.98, 32.21, 30.88, 30.78, 27.29, 26.85, 26.57, 25.83, 25.58, 23.38, 23.02, 21.36, 21.27, 17.45, 12.35. HRESIMS *m/z* 513.31673 [M + Na]^+^ (calcd for C_29_H_46_O_6_Na, 513.31866). Spectroscopic data obtained for DNER-4 was in agreement with previously reported literature values (Zhu et al., 2018)*.*


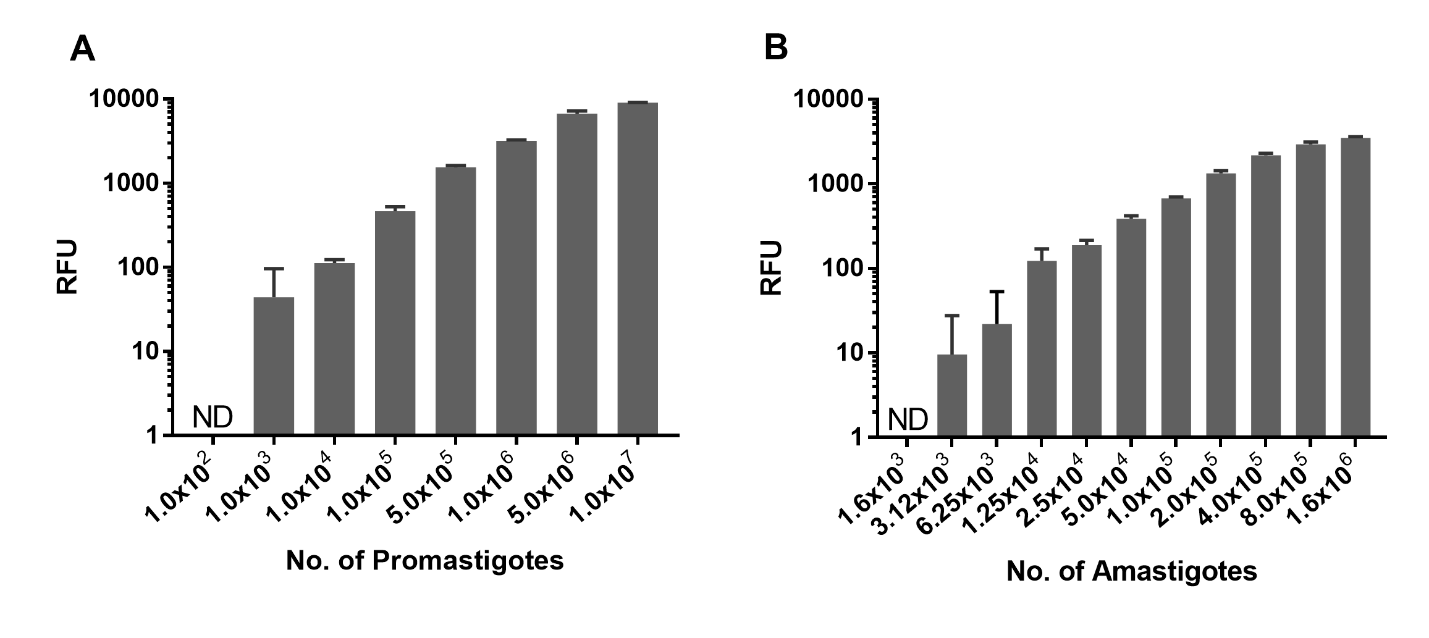


**Figure S1.** Validity of resazurin assay with *L. donovani* (A) promastigotes and (B) amastigotes. Strong correlation observed between Relative Fluorescence Units (RFUs) production with increasing parasite numbers as shown by Pearson’s r-analysis (*r* > 0.95 for promastigotes, and *r* > 0.92 for amastigotes). Data are reported as mean ± standard deviation (SD) of triplicate samples and with background subtracted. ‘ND’, significant RFUs were not detected after background subtraction.

**Table S1**

Host-directed selectivity index (SI) of anti-leishmanial drugs. All assays were run in triplicate and data (means ± SD) from two biological repeats. IC_50_: Inhibitory Concentration at 50%; LD_50_: 50% Lethal Dose for BMDMs.

| Compounds | Cytotoxicity LD_50_ (µM) against macrophages | Resazurin assay-based intracellular IC_50_ (µM) against *L. donovani* | Selectivity (LD_50_/IC_50_) |
| --- | --- | --- | --- |
|  |  |  |  |
|  |  |  |  |
| Amphotericin B | 38.5 ± 4.95 | 0.033 ± 0.011 | 1167 |
| Miltefosine | 62.5 ± 4.95 | 0.55 ± 0.15 | 114 |
| Paromomycin | >800 | 11.6 ± 0.42 | >68 |
| DNER-4 | 78.5 ± 9.2 | 3.2 ± 0.28 | 25 |
| AR-12 | 11.1 ± 2.5 | 1.44 ± 0.08 | 8 |


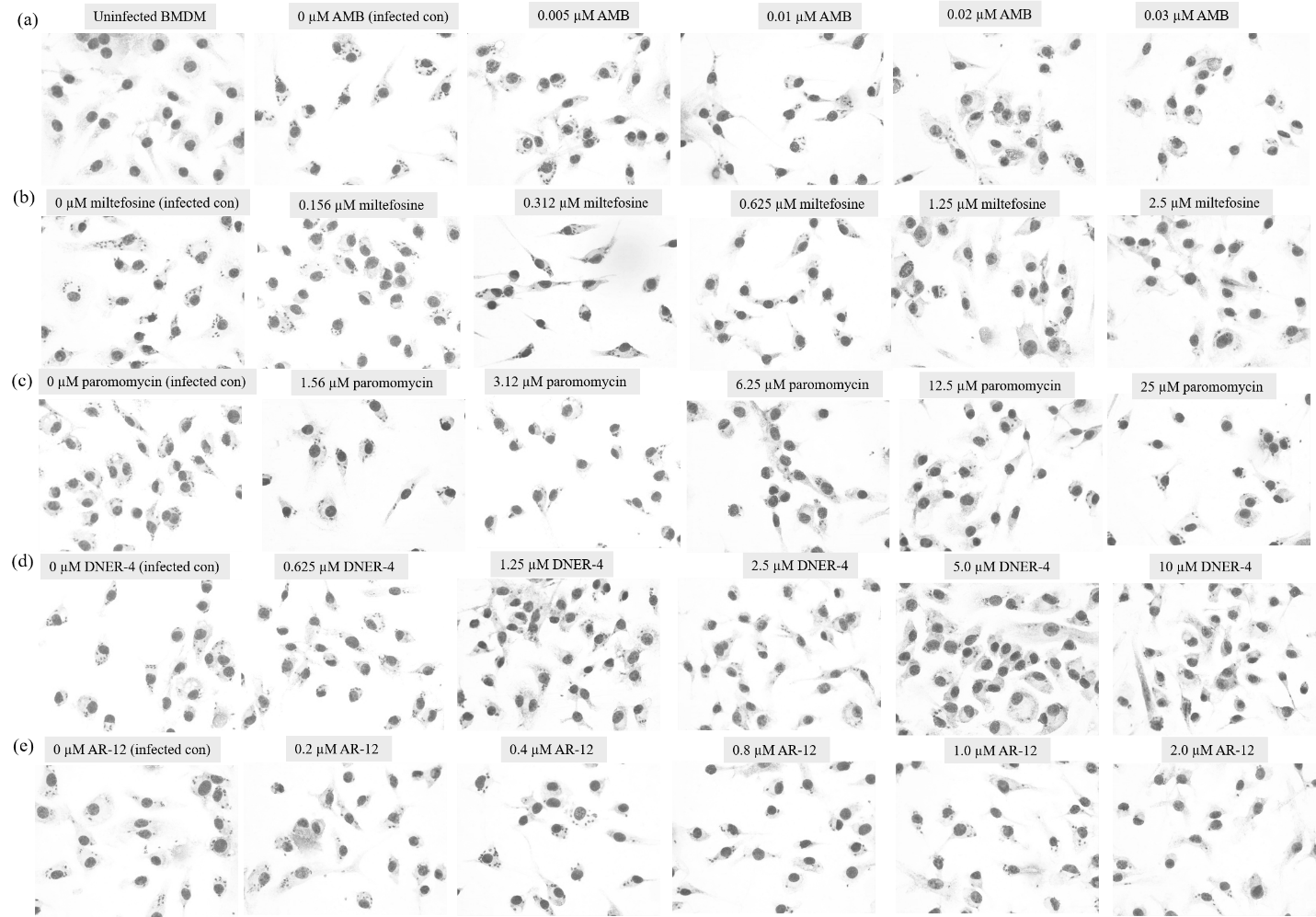


**Figure S2.** Image-based evaluation of intracellular anti-leishmanial effects of evaluated drugs. The smaller dark dots are the *L. donovani* amastigotes and larger is the BMDM nucleus (Giemsa-stained, 100x). Each panel is the representative images of infected macrophages treated with various concentrations of (a) amphotericin B (AMB), (b) miltefosine, (c) paromomycin, (d) DNER-4, and (e) AR-12.


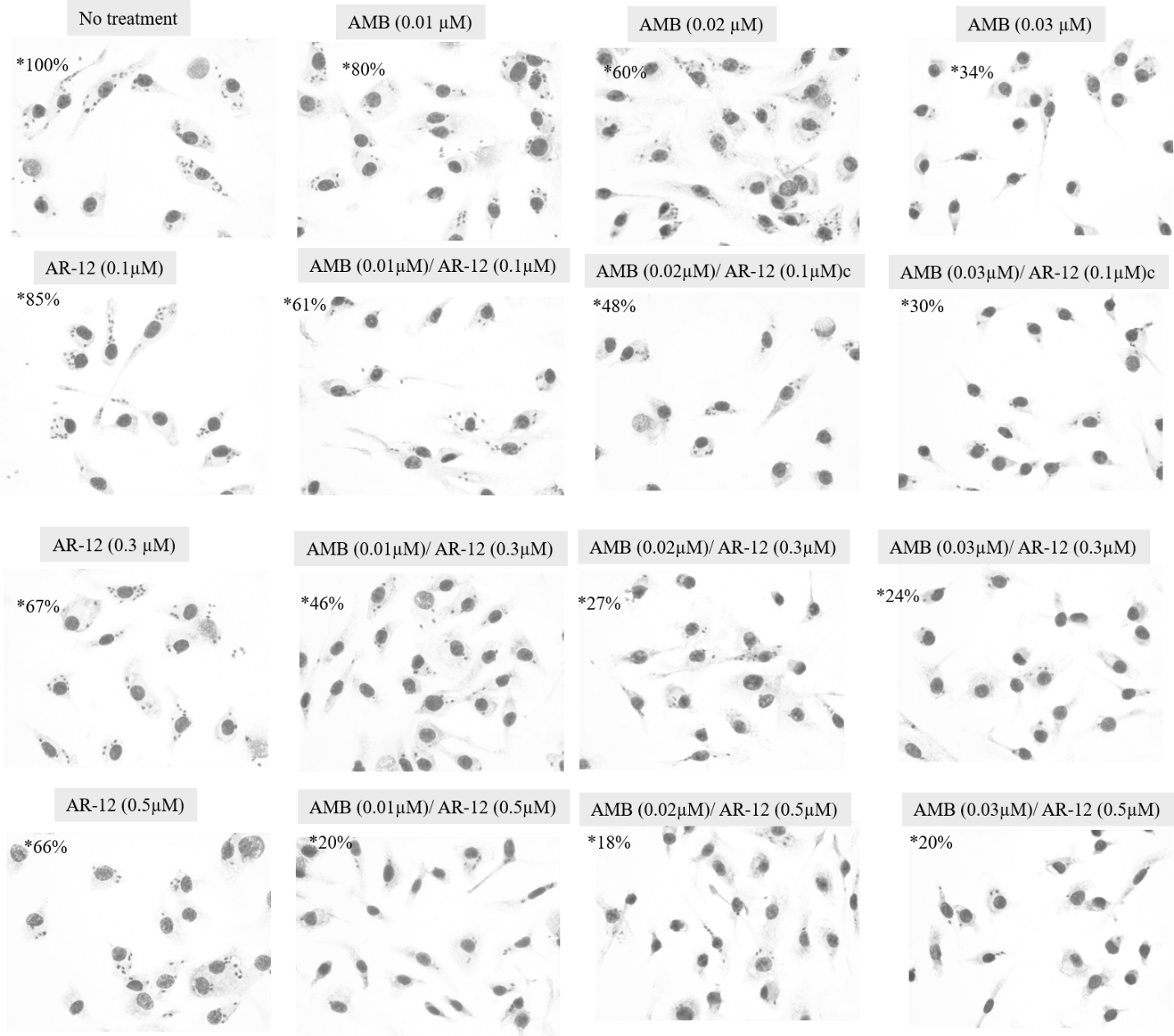


**Figure S3.** Image-based evaluation of intracellular anti-leishmanial combinatorial effects of amphotericin B (AMB) and AR-12. The smaller dots are the *L. donovani* amastigotes and larger is the BMDM nucleus (Giemsa-stained, 100x). ‘*’, % Parasite viability/100 BMDM nuclei. *L. donovani* infected BMDMs were treated with sub-optimal concentrations of drugs either in combination or monotherapy, and representative images are shown here.

**SI References**

do Nascimento, P.G., Lemos, T.L., Almeida, M.C., de Souza, J.M., Bizerra, A.M., Santiago, G.M., da Costa, J.G., Coutinho, H.D., 2015. Lithocholic acid and derivatives: Antibacterial activity. Steroids 104, 8-15.

He, X.L., Xing, Y., Gu, X.Z., Xiao, J.X., Wang, Y.Y., Yi, Z., Qiu, W.W., 2017. The synthesis and antitumor activity of lithocholic acid and its derivatives. Steroids 125, 54-60.

Zhu X., Qian B., Wei, R., Huang J., Bao H., 2018. Protection of COOH and OH groups in acid, base and salt free reactions. Green Chem 20 (7), 1444–47.
